# Challenges and opportunities in the recovery of gold from electronic waste

**DOI:** 10.1039/c9ra07607g

**Published:** 2020-01-27

**Authors:** Mudila Dhanunjaya Rao, Kamalesh K. Singh, Carole A. Morrison, Jason B. Love

**Affiliations:** Department of Metallurgical Engineering, Indian Institute of Technology (Banaras Hindu University) Varanasi-221005 India kksingh.met@iitbhu.ac.in; EaStCHEM School of Chemistry, University of Edinburgh, Joseph Black Building, The King's Buildings David Brewster Road Edinburgh EH9 3FJ UK carole.morrison@ed.ac.uk jason.love@ed.ac.uk

## Abstract

Rapid global technological development has led to the rising production of electronic waste that presents both challenges and opportunities in its recycling. In this review, we highlight the value of metal resources in the printed circuit boards (PCBs) commonly found in end-of-life electronics, the differences between primary (ore) mining applications and secondary (‘urban’) mining, and the variety of metallurgical separations, in particular those that have the potential to selectively and sustainably recover gold from waste PCBs.

## Abstract 2

Rapid global technological development has led to the rising production of electronic waste that presents both challenges and opportunities in its recycling.

## Graphical abstract

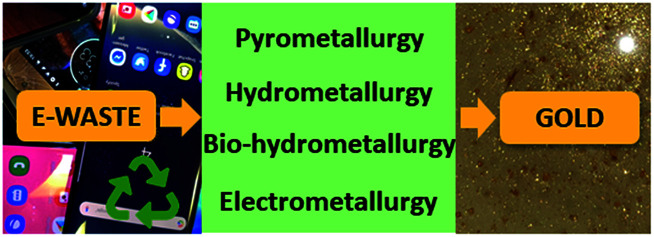

The rapid global rise in technology, tied in with consumer pressures for upgrades in functionality and design, has generated advanced electrical and electronic equipment with short lifespans. A consequence of this is the production of electronic waste (e-waste) which, in 2018 amounted to 50 million tonnes,^1,2^ with a projected annual growth of 3–5%, three times more than for other waste streams.^3^ Reports on recycling rates vary, with estimates of around 20–30%.^1,4^ It is estimated that more than 70% of globally produced waste electronics and electrical equipment (WEEE) enter China, Africa and India for reprocessing, much of it illegally, and often using crude, hazardous and inefficient processes.^5,6^ Dumping and incinerating large amounts of WEEE has severe impact on human life and the environment,^7^ as it leads to the release of toxic heavy elements such as lead, mercury, chromium, nickel, beryllium, arsenic and antimony into the air, soil and water cycles.^8^

An end-of-life printed circuit board (PCB) may contain up to 60 different chemical elements,^9^ and have a metal content as high as 40% by weight,^10^ so should be viewed as a valuable secondary source of precious and base metals. The metal content of a PCB is typically ten to a hundred times higher than that of conventionally mined ores.^11^ It is estimated that recycling one ton of mobile phones could produce on average 130 kg of copper, 3.5 kg of silver, 0.34 kg of gold and 0.14 kg of Pd.^12^ On this basis, the global e-waste management market is projected to produce an annual revenue of USD 62.5 billion by the end of 2020.^2,13^ With an estimated 97% of the world population owning a mobile phone,^14^ it can be viewed as a plentiful feedstock for a recycling process. As such, the treatment of e-waste not only helps minimise the environmental impact of our technology-driven society by reducing pollution and energy demands compared to conventional mining practice,^15^ it also presents economic drivers for wealth creation and circular economies.^16–21^

In this review, we outline some of the latest chemical approaches that have been reported for the recovery of gold from discarded mobile phones and other WEEE.^22,23^ Gold is the most valuable component of e-waste, with estimates for its consumption to fuel our technology-driven society at 263.3 MT per year.^7,24^ We provide an overview of metal concentrations that are present in waste PCBs from end-of-life mobile phones, analyse the different pre-treatment steps that can be used to separate the metallic and non-metallic components of PCBs, and highlight various metallurgical methods for the extraction of gold from waste PCBs. For this latter aspect, we focus on methods in the primary research literature for which an understanding of the chemical mode of action has been developed; as such, a detailed analysis of the patent literature is not in the scope of this review.

## Gold recovery from printed circuit boards

A typical PCB comprises 40% metals, 30% plastics and 30% ceramics,^5,10^ with the metal fraction comprising 10–27% Cu, 2–8% Al, 1–4% Pb, 1–8% Fe, 1–6% Sn, 0.2–3.6% Ni, 0.1–1.5% Zn and <0.1% precious metals.^25–30^ These data were typically obtained by milling the waste PCBs and then leaching the powder with *aqua regia* (a 1 : 3 mixture of nitric and hydrochloric acid), or alternatively hydrochloric acid followed by *aqua regia*. The levels of precious metals in electronic waste vary considerably, from 10–1600 ppm of Au, 200–20 000 ppm of Ag, and 5–970 ppm of Pd, but in most cases exceed those expected in conventionally mined ores; a rich gold-containing ore is typically 0.0018 wt% (18 ppm) of gold and a typical silver bearing ore contains 0.085% (85 ppm) of silver.^31^ It is also apparent that the concentration of precious metals found in electronic waste is dependent on the age of the device; the thickness of gold contacts halved from *ca.* 1.0 μm in devices manufactured in the 1980s to 0.6–0.3 μm for those made in the 2000's.^11^

### Pre-separation treatment of e-waste

**Fig. 1:**
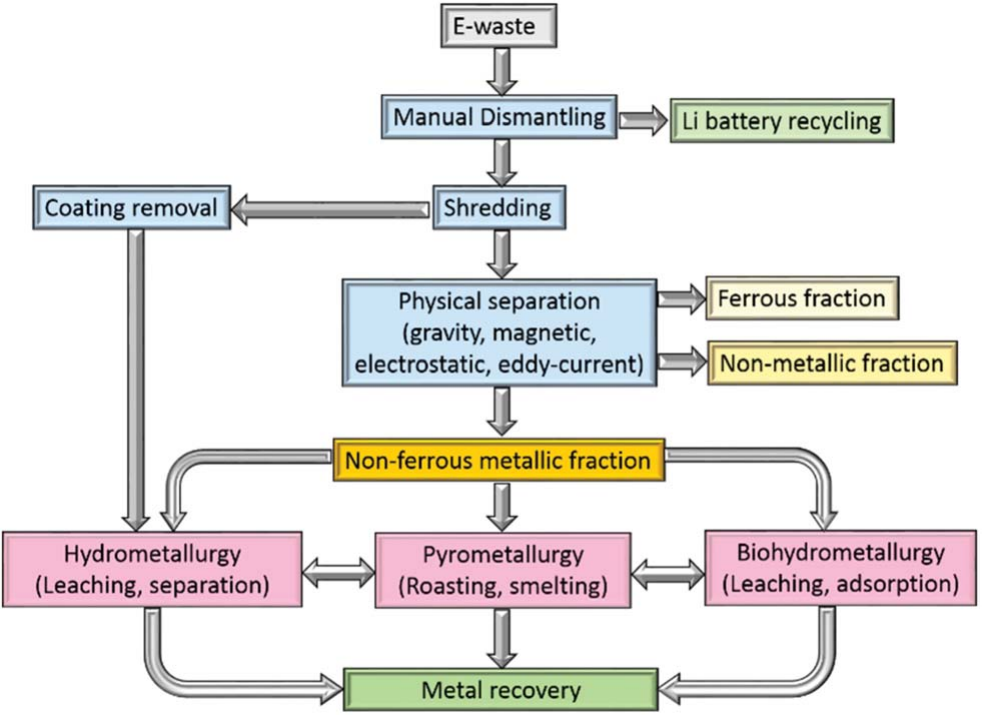
Overview of stages involved in metal recycling from electronic waste.

The processing of e-waste typically begins with a manually intensive dismantling phase, during which circuit-board components and the lithium battery are removed for recycling elsewhere (Fig. 1). The PCBs are subsequently graded according to their metal : plastic ratio and shredded, typically into 1.0 cm^2^ pieces. The shredded PCBs need to be separated into metallic (ferrous and non-ferrous), and non-metallic (polymer and ceramic) components and a broad range of methods have been identified for this purpose, including mechanical crushing, followed by separation using gravity, electrical conductivity and magnetism, as well as delamination using organic solvents.

Multistep crushing provides high shear forces that detach the metals from PCBs, with copper wires and joints particularly prone to disintegration. Whilst this is a reasonably energy efficient process,^32^ crushing alone cannot typically yield the fine particles that are required to improve metal recovery rates.^33^ Consequently, the output from a crusher is typically subjected to a further mechanical separation step. One option uses gravity separation in which waste PCBs are milled to a particle size to below 5 mm, allowing the lighter (non-metallic) fraction to be separated from the heavier (metallic) fraction.^34^ Alternatively, electrostatic methods separate metallic and non-metallic components based on their electrical conductivity or resistivity. Practical difficulties exist, however, such as the treatment of the so-called middling product (a granular intermediate product of conducting and non-conducting materials) and the removal of non-conducting materials, but both of these issues have largely been addressed by the development of a two-roller type corona-electrostatic separator of high productivity rate and good energy efficiency.^35^ Moreover, the process does not evolve wastewater or dust during the process, which is an advantage over other separation methods.^33^ Alternatively, the eddy current separator is widely used,^10^ which exploits rare-earth permanent magnets to separate non-ferrous metals from the waste once all ferrous metals have been removed.^36^

As a chemical technique, delamination of the interwoven metallic and non-metallic layers in a PCB, which are bonded together with halogenated epoxy resin, can be undertaken. The resin can be dissolved using organic solvents such as dimethyl sulfoxide,^37^*N*,*N*-dimethyl pyrrolidone,^38^ dimethylformamide, or dimethylacetamide. Amide-based solvents have been found to give superior results primarily due to their lower evaporation rates.^39,40^ Ionic liquids, such as 1-butyl-3-methylimidazolium chloride have also been shown to dissolve up to 90% of the bonding resin.^41^

## Chemical techniques for gold recovery

### Pyrometallurgy

Pyrometallurgical processes include roasting, in which compounds are converted at temperatures just below their melting points, and smelting, which involves higher temperatures to completely melt the material which is then separated into two liquid layers, one of which contains the metals for further refining.^5,42,43^ Oxygen-enriched air and fuel may be injected into the molten bath through a lance to oxidise and remove any volatile components present, while passage of an electric current in electrometallurgy processes acts to dissociate any metallic compounds present in the electrolyte and deposits the metal at the cathode. Pyrometallurgy offers the advantage that a pre-treatment step beyond unit dismantling and shredding of the components is rarely required.^31,44^ The output from the smelter for electronic waste is best described as a copper bullion, due to the high copper content found in PCBs. The copper can be separated by leaching and recovered by electrowinning, leaving a residue of precious metals for further refining (see later).

The smelting process is energy-intensive, but the overall reliance on fossil fuels (*e.g.* coke) can be partially offset by exploiting the plastic content of PCBs as both a fuel and a reducing agent in the smelter.^45^ However, as PCBs contain halogenated flame retardants this leads to the formation of furans and dioxins, which, along with the creation of volatile metals and dust gives rise to environmental challenges.^25^ While pyrometallurgical recycling processes are a cost-effective solution for electronic-waste recycling due to economy of scale and ability to deal with a broad range of scrap materials with minimal pre-processing, they carry a large environmental burden. This type of recycling also displays poor selectivity for individual metals, meaning that multiple stages are required to recover metals in their pure elemental form.^5^

Recently, an optimised process for the recycling of complex metallic materials such as waste PCBs was developed, based on a top-blown rotary converter smelter, with an oxygen-propane lance and a 360° rotating chamber that tilts to different angles to allow poring of slag and casting fractions.^46^ Pyrolysis is introduced as a pre-processing method for enhanced separation of the non-condensable gas and liquid fractions and solid residue, with the resulting solid material making the separation of metals, glass fibre and organic fractions easier and consequently the recycling of each portion more viable. Umicore's Hoboken plant in Belgium has developed an advanced process which includes the recovery of copper and precious metals, along with a waste gas and water utilization system.^25^ Furthermore, a new process has been introduced for the simultaneous extraction of precious metals from waste mobile phone PCBs and honeycomb-type autocatalysts by smelting with industrial-waste copper slag. This process is simpler than conventional pyrometallurgical process as the addition of any external collectors are not required.^47^

### Hydrometallurgy

Metal separation and recovery using hydrometallurgical processes have lower capital cost and environmental impact than pyrometallurgy, and offer greater scope for selective metal recovery which greatly simplifies the production of highly purity metals. In conventional mining, hydrometallurgy is more suited to recovering metals from lower grade, mixed-metal ores than can traditionally be handled by pyrometallurgical routes.^48,49^ However, challenges arise from the complexity of the feed stream, the need for strong acids in leaching processes, and the need to minimise the losses of the organic solvents and chemical reagents during the separation processes.

**Fig. 2:**
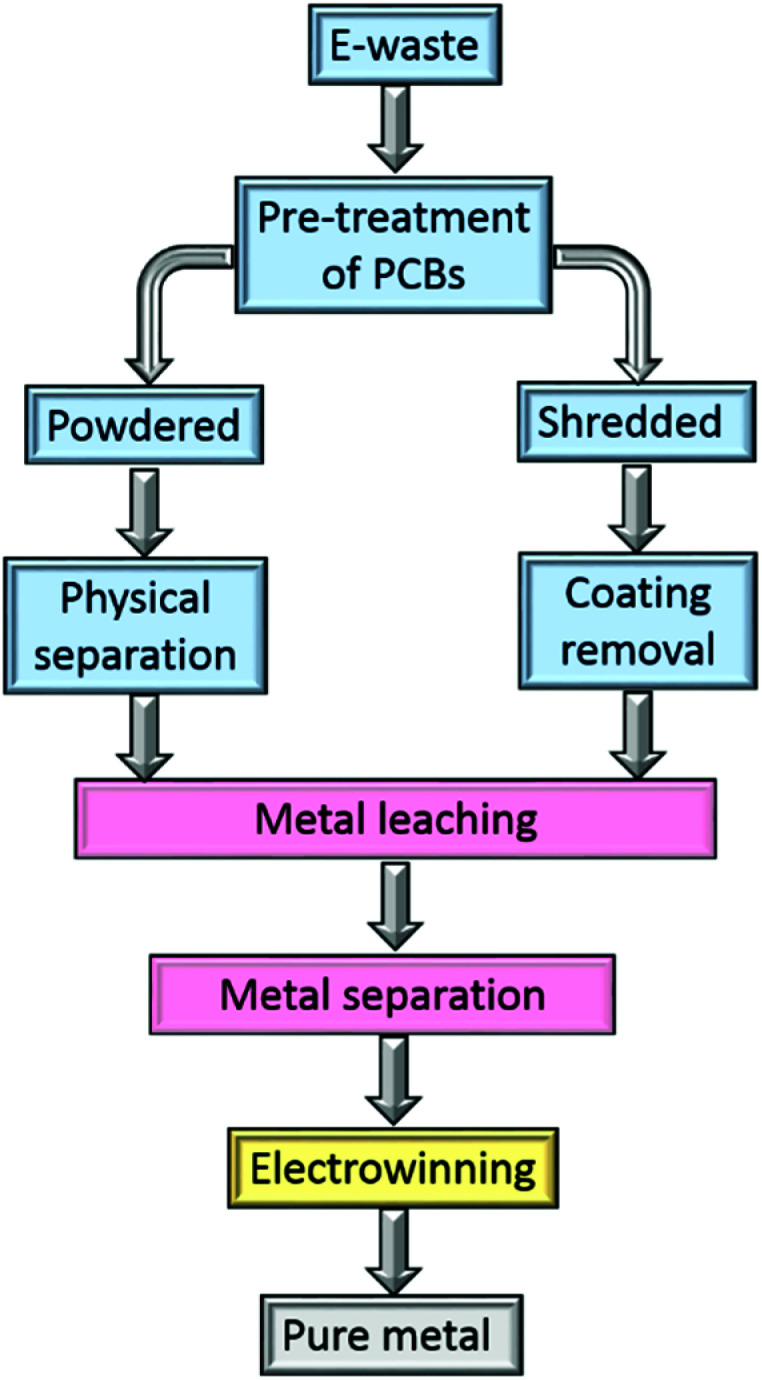
Main stages in a hydrometallurgical process to recover metals from waste electronics.

In the hydrometallurgical recovery of gold from electronic waste (Fig. 2), the PCBs are leached by a suitable lixiviant, usually after chemical pre-treatment.^50^ The resultant pregnant leach liquor then undergoes a separation step to obtain single metal streams from which pure metals are obtained, for example by electrowinning.

#### Leaching

The selection or development of a leaching process plays a pivotal role in hydrometallurgy, as it must provide efficient dissolution of metals from PCBs and deliver them in a suitable form for the separation step. Importantly, metals such as gold are in their elemental form in electronic waste, so will need to be oxidised during dissolution, *e.g.* to Au(i) or Au(iii); this contrasts with conventional mining from ores in which metal cations are already present as oxides or sulfides.

Cyanide is a cheap but highly toxic reagent that is very effective in leaching gold from low-grade minerals as the water-soluble cyanoaurate [Au(CN)_2_]^−^ (eqn (1)).

1 4Au_(s)_ + 8CN^−^_(aq)_ + O_2(g)_ + 2H_2_O_(l)_ → [Au(CN)_2_]^−^_(aq)_ + 4 OH^−^_(aq)_

The well-documented toxicity and environmental concerns around the use of cyanide in the gold mining industry^12^ has led to the adoption of the International Cyanide Management Code, a voluntary program intended to reduce the potential exposure of workers and local communities to the harmful effects of cyanide. It is estimated that cyanide leaching is used in around 90% of gold production from primary ores,^51^ and a similar story emerges for e-waste recycling, with cyanide reported as the principal gold leaching agent currently in use in China.^52^ While cyanide leaching from minerals is very effective, it was reported that just 60% of the gold could be recovered from pulverised waste PCBs using a commercial cyanide leachant.^12^

Much work has been undertaken to develop alternatives to cyanide leaching.^43,53–55^ Thiocyanate has been found to leach gold as [Au(SCN)_2_]^−^ or [Au(SCN)_4_]^−^ in the presence of an Fe(ii)/Fe(iii) catalyst. It can act as a lixiviant over a wide pH range and is reported to be partly recyclable, but its use is restricted to higher temperatures.^55,56^ Similarly, thiosulphate leaching (eqn (2)) has been exploited in gold leaching and, although relatively cheap and less toxic than cyanide, it is also less efficient and significant problems exist due to complex reaction kinetics; even with the addition of oxidisers such as H_2_O_2_, the level of gold recovery by thiosulfate can be lower than 15%.^12,57,58^

2 4Au_(s)_ + 8S_2_O_3_^2−^_(aq)_ + O_2(g)_ + 2H_2_O_(l)_ → 4[Au(S_2_O_3_)_2_]^3−^_(aq)_ + 4OH^−^_(aq)_

Thiourea has also been investigated as a leachant, which, in the presence of iron sulfate, creates the water-soluble cationic gold(i) complex Au[SC(NH_2_)_2_]_2_^+^ (eqn (3)).^59^ A potential drawback in thiourea leaching is that the high abundance of copper in PCBs increases the rate of thiourea decomposition to elemental sulphur, which passivates the gold surface.^60^ Even so, it was reported that thiourea could extract up to 90% of the gold from mobile phone PCBs.^61^

3 Au_(s)_ + 2SC(NH_2_)_2(aq)_ + Fe^3+^_(aq)_ → Au{SC(NH_2_)_2_}_2_^+^_(aq)_ + Fe^2+^_(aq)_

**Fig. 3:**
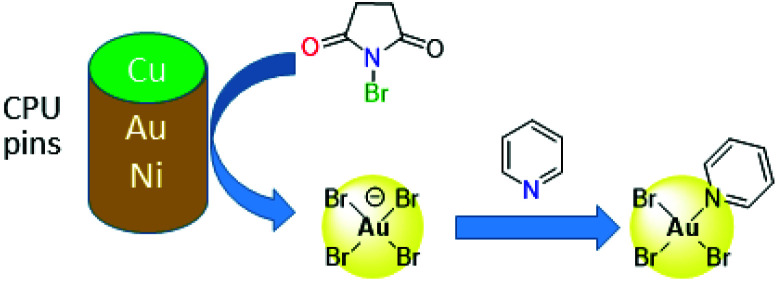
Leaching of gold from e-waste using *N*-bromosuccinimide/pyridine mixtures.

Other alternatives to cyanide include halide leaching, whereby the strong oxidants Cl_2_ or Br_2_ are generated *in situ*, either electrochemically or by reaction between sulfuric acid and hydrochloric or hydrobromic acid or a halide salt, with the latter reported as effective in copper leaching.^47,62,63^ Other oxidants such as O_2_, Cu(ii), Fe(iii) or nitric acid are also used in addition to halides,^53^ and the non-toxic ammonium persulphate is reported to have greater lixiviant properties than potassium or sodium persulphate.^64^ More recently, synergistic mixtures of *N*-bromosuccinimide (NBS, a strong oxidant) with pyridine (py, an effective complexing ligand) have been found to offer a cheap and low-toxic route to selective gold leaching (Fig. 3).^65^ Initial oxidation of gold by NBS from the surface of CPU pins occurs to form low concentrations of bromoaurate [AuBr_4_]^−^, which is stabilised by the formation of the neutral complex AuBr_3_(py) by reaction with pyridine; about 90% of the gold is leached using this mixture compared with *ca.* 40% recovery of other metals found in waste PCBs.

Dissolution of elemental gold was also accomplished using dimethylformamide solutions of pyridine-4-thiol as a reactive ligand and hydrogen peroxide as an oxidant.^66^ In this case, the thiol isomerises to the thione (PS) which interacts with Au(0) at the surface. Oxidation to Au(i) by H_2_O_2_, with complementary oxidation of the ligand, ultimately to sulphuric acid, results in [Au(PS)_2_]_2_[SO_4_] as the final gold product in solution.

*Aqua regia* has received attention in recent years in the leaching of gold due to its complete dissolution and fast rates.^67,68^ While its strongly oxidising and corrosive nature render it unsuitable for full-scale operations,^61^ it is a suitable leachant for use in fundamental research. The nitric acid acts as a powerful oxidising agent to form Au^3+^ ions, while the hydrochloric acid provides a large excess of Cl^−^ ions to form H[AuCl_4_] (eqn (4) and (5)).

4 Au_(s)_ + 3HNO_3(aq)_ + 4HCl_(aq)_ ⇌ H[AuCl_4_]_(aq)_ + 3NO_2(g)_ + 3H_2_O_(l)_

5 Au_(s)_ + HNO_3(aq)_ + 4HCl_(aq)_ ⇌ H[AuCl_4_]_(aq)_ + NO_(g)_ + 2H_2_O_(l)_

As an oxidising acid, HNO_3_ has been shown to act as a two-stage leachant, selectively dissolving copper, nickel and gold.^69^ Initially, a dilute HNO_3_ (0.1 M) leach step results in suppression of copper leaching but enhanced nickel leaching due to its higher chemical reactivity; increasing the concentration of HNO_3_ (to 1.0 M) results in high recovery of both copper and gold (98%). A solvent extraction step (using a commercial oxime-based reagent) separated this latter mixed-metal stream.

The oxidation of waste PCBs using supercritical water (*T* > 647 K, *P* > 218 atm) and sodium hydroxide as a first step for the removal of harmful organic species originating from the degradation of toxic matter (*e.g.* brominated flame retardants) from waste PCBs has been reported.^70^ This process was later modified to enhance the leaching of copper along with precious metals gold, silver and palladium.^71^ In this latter case, HCl was used as the leachant for the initial recovery of copper, followed by iodine–iodide (oxidant and complexing agent, respectively) for subsequent dissolution of the precious metals.

#### Adsorption and precipitation

Adsorption and cementation are prominent techniques for the recovery of gold from low concentration cyanide solutions derived from commercial mining.^72^ Adsorption methods are cheap and simple to operate and typically involve adsorbing the cyanoaurate [Au(CN)_2_]^−^ on activated carbon particles, which due to their large size can be readily separated from the leach liquor by filtration. The gold is then subsequently released from the loaded carbon by heat (*e.g.* using a smelter) or pH control (*e.g.* on contact with sodium sulfide).^73^ These methods are referred to as Carbon-in-Pulp (CIP) methods, with Carbon-in-Leach (CIL) and Carbon-in-Column (CIC) as other variants on this theme.^74^ Cementation methods involve passing the gold leachate solution through a bed of metal shavings or powder. The Merrill–Crowe process uses zinc cementation in which the filtered cyanide solution is passed through deaerating columns to remove the oxygen before adding zinc dust to reduce and precipitate the gold (eqn (6)).^73^ The precipitated gold is then recovered by filtration, mixed with fluxes (borax, silica, or sodium carbonate) to bind with impurities, and smelted to form bars which are then sent for the further refining processes.

6 Zn_(s)_ + 2Au(CN)_2_^−^_(aq)_ → 2Au_(s)_ + Zn(CN)_4_^2−^_(aq)_

**Fig. 4:**
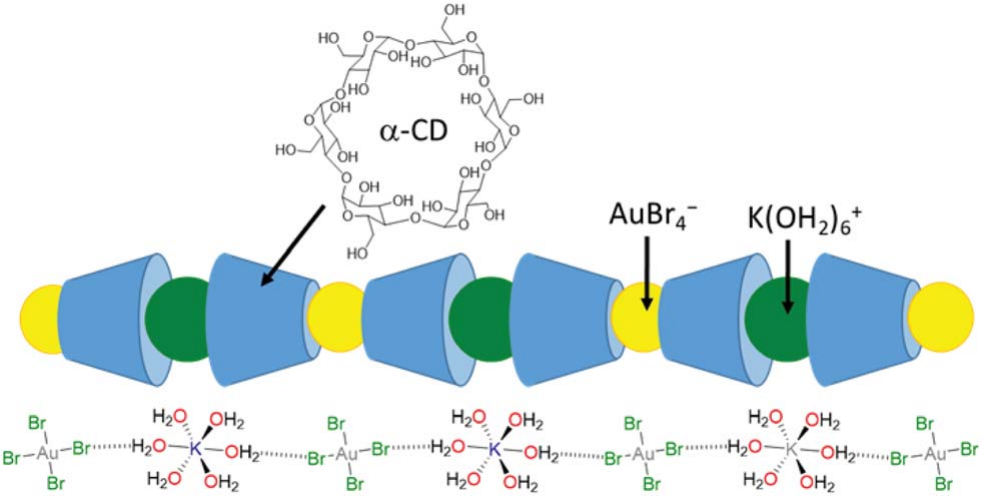
Selective precipitation of gold as a KAuBr_4_/α-cyclodextrin assembly.

The selective recovery of gold (as K[AuBr_4_]) has been demonstrated through its co-precipitation with α-cyclodextrin (Fig. 4).^75,76^ In this case, the insoluble 1D supramolecular polymer {[K(OH_2_)_6_][AuBr_4_](α-cyclodextrin)_2_}_*n*_ is formed in which precise molecular recognition between [AuBr_4_]^−^ and α-CD occurs; the axial orientation of the anion within the α-CD cavity favours specific second-sphere electrostatic and hydrogen bonding interactions between the anion and K(OH_2_)_6_^+^ cation. Life-cycle analysis indicated that application of this technology could significantly reduce the current environmental impact of gold nanoparticle synthesis.^77^

**Fig. 5:**
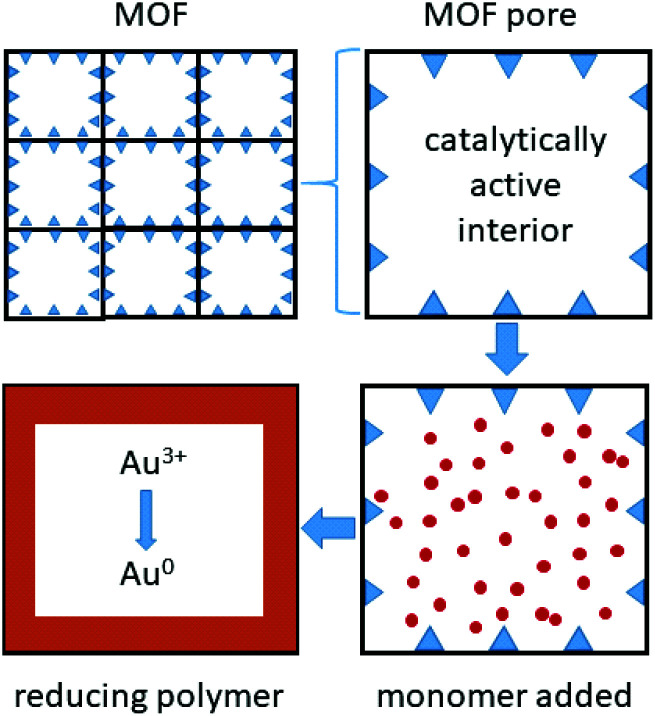
Metal organic frameworks, with catalytically active metal sites that bind and polymerise monomers within the pores, forming short redox-active polymer chains that bind and reduce gold.

Metal organic framework (MOF) materials also appear promising for gold recovery (Fig. 5). The large pores in the framework Fe-BTC (where BTC = 1,3,5-benzenetricarboxylate), have been lined with short redox-active poly(*meta*-aminophenol) chains that bind and reduce gold complexes formed in a solution similar to that expected from an *N*-bromosuccinimide/pyridine leached solution from waste PCBs (Fig. 3).^78^

**Fig. 6:**
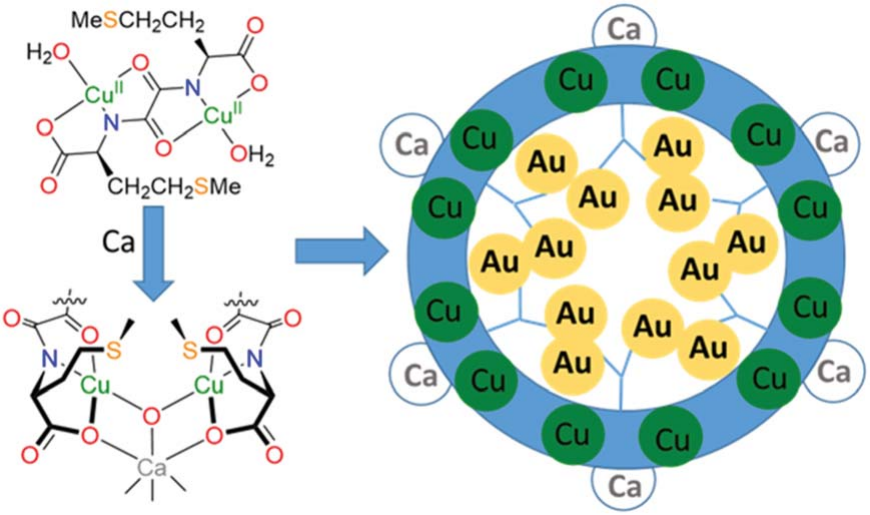
Metal-templated formation of MOFs with thioether-decorated pores for gold adsorption.

MOFs that arrange sulphur-donor atoms within their porous cavities have been prepared and exploited for the adsorption of gold from water solutions (Fig. 6).^79^ These MOFs were constructed from copper complexes of chiral bis(l-methionine)oxalamide ligands that, on addition of Ca(ii) ions, formed porous solids with hexagonal channels of *ca.* 0.3 nm diameter. Soaking these materials in water solutions of AuCl_3_ or AuCl resulted in the formation of the thioether complexes of gold (RS)AuCl and (RS)AuCl_3_ within the porous channels, with aurophilic interactions evident between the Au(i) centres. Gold recovery of 90% from acidic leach solutions from waste PCBs was achieved using polyaniline films to reduce the gold to its elemental state;^80^ the polymer could subsequently be regenerated, offering potential for efficient gold recovery without the use of extractant reagents or external energy input. Similarly, a simple and efficient water-soluble fluorescent conjugated polymer (poly(2,5-bis(polyethylene glycol oxybutyrate)-1,4-phenylethynylene-*alt*-1,4-phenyleneethynylene; PPE-OB-PEG)) was prepared from commercially available 1,4-diethynylbenzene and PEG-2000, for selective detection and extraction of Au(iii) cations in e-waste;^81^ an 80% extraction efficiency was reported through the selective formation of alkynyl–Au bonding interactions.

Ion-exchange process involving the adsorption of gold from a leach solution using a cation-exchange polymer resin have been utilised for gold recovery from e-waste.^82^ The basic process is similar to CIC except that the elution (metal stripping) stage from the loaded resins does not require high temperatures. Interestingly, 3D printed meshes and columns of nylon-12 in which amide-group scavengers (see later) are intrinsically present have shown to selectively adsorb 78% of [AuCl_4_]^−^ from PCBs leached by *aqua-regia*; multiple wash steps using dilute nitric acid resulted in 99% gold recovery.^83^ An advanced technology for the selective recovery of gold from waste electronics using electro-generated chlorine gas as an oxidant in an HCl leach stream has been proposed.^84^ In common with other studies, the copper was separated first using 2 M HCl, leaving a residue from which gold was recovered (99.99%) by ion-exchange chromatography.

#### Solvent extraction

An alternative technique for gold recovery from the leach liquor is solvent extraction, a scalable technique for the selective separation of a particular metal from a mixed-metal feedstock.^49,85^ This is particularly important for the recycling of waste electronics, where the concentrations of base metals far outweigh the concentrations of gold and other precious metals. The success of the solvent extraction process resides with the efficiency and selectivity of the metal extractant, and ensuring that good separation is achieved between the two phases. Selectivity is achieved through coordination and supramolecular chemistry principles by designing ligands that can differentiate between the different metal ions on the basis of size, charge and shape.^49,86,87^

The solvent extraction of halometalates such as [AuCl_4_]^−^ from halide leach solutions derived from gold ores is carried out commercially using simple solvents such as methyl isobutyl ketone (MIBK), dibutyl carbitol (DBC), or 2-ethyl hexanol (2-EH). However, selectivity, safety, and mass balance issues are evident in separations using these solvents and the chemical modes of actions remain poorly understood.^88^

**Fig. 7:**
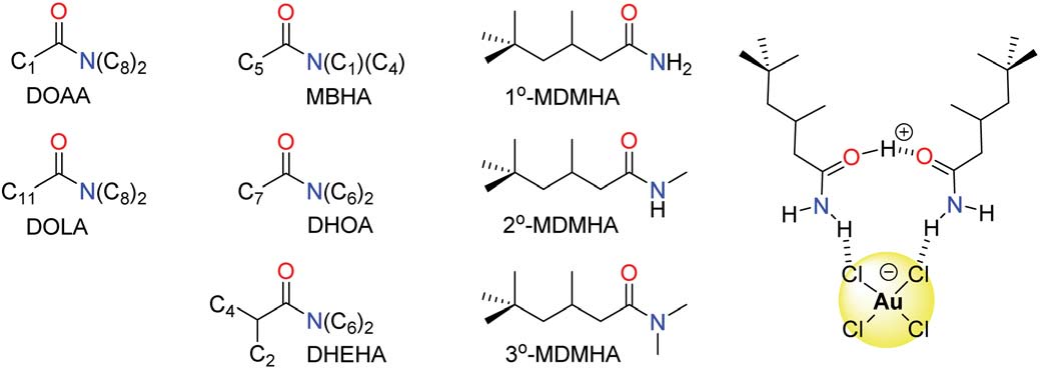
Amide reagents exploited for the recovery of gold by solvent extraction.

Organic amides have been long studied as reagents for selective gold recovery by solvent extraction (Fig. 7). Tertiary amides such as DOAA and DOLA show good selectivity for gold over other precious metals such as Pd, Pt, and Rh and base metals such as Fe, Cu, Ni, and Zn.^89^ However, third phases are often formed and the strip stage of the solvent extraction process can require the use of thiourea, thus affecting mass balance. The use of unsymmetrical substituents in MBHA enhance extraction efficiency, and slope analysis (log *D vs.* log L, where *D* = distribution coefficient and L = ligand) suggested the formation of complexes of the stoichiometry HAuCl_4_(amide)_2_ in the organic phase (Fig. 7).^90^

More recently, the simple primary amide 1°-MDMHA was shown to achieve the selective separation of gold as [AuCl_4_]^−^ from an aqueous mixed-metal solution of composition similar to that obtained from HCl leaching of waste PCBs.^91^ Protonation of MDMHA plays a crucial role in the selective extraction of gold, as combination of the protonated and neutral amide with [AuCl_4_]^−^ through hydrogen bonding and electrostatic interactions creates a neutral assembly which is transported into the organic phase (Fig. 7). Maximum extraction of gold (*ca*. 80%) was observed at 2.0 M HCl, a point at which the extraction of the other metal ions (*e.g.* Fe, Cu, and Zn) typically found in a PCB was very low. The concentration of extractant needed was low (0.1 M) which, along with the observation that the back transfer of [AuCl_4_]^−^ into a clean aqueous solution can be achieved using just water, is in stark contrast with commercial gold extractants such as MIBK and DBC.

Subsequent studies on secondary (2°-MDMHA) and tertiary amide (3°-MDMHA) analogues of 1°-MDMHA (Fig. 7) have shown that the 2° and 3° amides are stronger extractants for gold from single-metal solutions, yet show poor extraction efficiency from a mixed-metal solution representative of e-waste.^92^ In these cases, the presence of high concentrations of other metals such as Cu, Fe, and Sn cause the formation of viscous third phases (insoluble in both aqueous and organic phases); the use of a more polar organic phase circumvents third-phase formation, but with a loss in selectivity for gold. The identities of the species formed in the organic phase was probed using spectroscopic, diffraction, and computational methods, and further highlighted that transport of the proton into the organic phase by the amide as H(L)_2_^+^ (where L = amide) is important, and that little or no water is involved in the organic-phase assembly process.

**Fig. 8:**
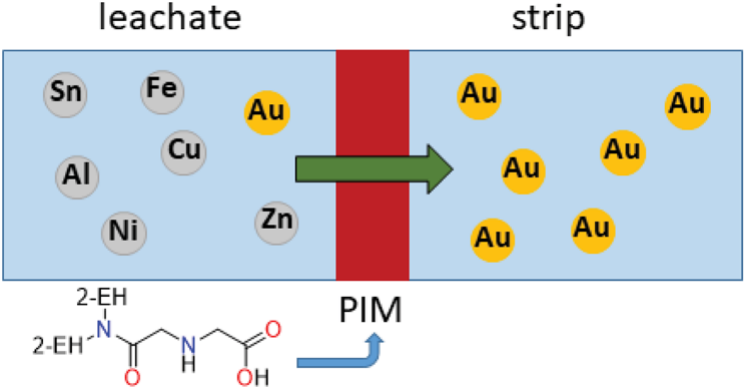
Transport of Au from an aqueous leach solution into and aqueous strip solution with selectivity provided by the extractant-embedded polymer inclusion membrane (PIM).

Recently, a polymer inclusion membrane (PIM) into which an aminocarbonylmethylglycine extractant is embedded was shown to selectively separate gold from an *aqua regia* e-waste leach solution.^93^ In this case, Au transport is achieved between the leach solution and an aqueous strip solution containing thiourea *via* the extractant-embedded PIM (Fig. 8), thus negating the need for an organic solvent in a liquid–liquid solvent extraction system.

#### Electrochemistry

**Fig. 9:**
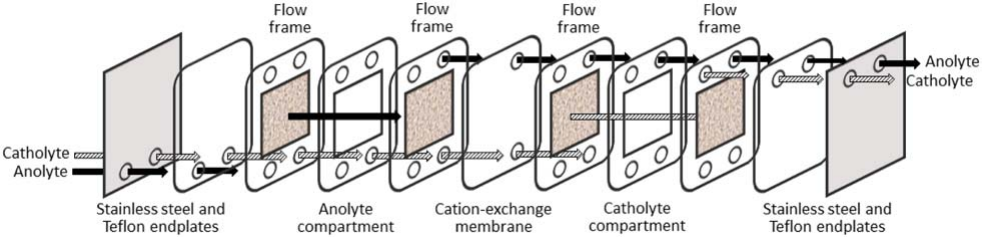
Schematic diagram of a flow-through electrogenerative reactor (modified from ref. 94).

Several electrochemical separations have also been developed, including a process to recover gold from a cyanide leachate solution using a highly porous glassy carbon cathode;^94^ 99% of gold was recovered in 1 h due to the electrode's large porous surface area, high void volume, rigid structure and low resistance to fluid flow (Fig. 9). This process was improved by purging the electrochemical cell with nitrogen gas to remove any dissolved oxygen which was known to inhibit the deposition of gold,^15^ allowing gold recovery from solutions of low concentration (*ca.* 100 mg L^−1^). Cyclic voltammetry experiments applied to *aqua regia* leach solutions from PCBs have demonstrated that pure gold can be electro-deposited directly from solution without interference from the other metal ions present.^95^ Gold extraction levels of 99.9% were achieved using gold electro-deposition from cyanide leach solutions with a zinc powder cathode system.^72^

Removing the dominant copper metal from the PCB as a first step can allow the precious metals to be processed in a more efficient manner. The use of an acidic ferric chloride solution, along with simultaneous electrowinning, results in a higher weight percentage of gold in the remaining solid residue.^96^ In this instance copper recovery was high (99%), and electrochemical recovery of gold was more efficient as the residues were 25 times more concentrated in gold compared to the original PCB samples. Similarly, gold was leached from the solid residue with HCl/H_2_O_2_, followed by electrowinning, to generate a high purity (99.99%) gold deposit.^97^ A complete process has been developed for the processing of waste PCBs which also favours stripping out the dominant metals as a first step.^98^ Iron and aluminium were removed first using magnetic and eddy current separation, followed by dissolution of the copper (by ammonium sulfate leaching, solvent extraction and electrowinning) to leave a solid residue (*ca.* 2 wt% of the original material) which was leached using *aqua regia* and the gold extracted using tetraoctylammonium bromide in toluene. The recovered gold was then converted into nanoparticles (97 wt%) in the presence of dodecanethiol and sodium borohydride to increase the value of the final product.

### Biohydrometallurgy

Until relatively recently biohydrometallurgy was largely confined to just two industrial applications: the processing of low-grade copper ores,^99^ and the recovery of ultrafine gold particles from refractory ores that are resistant to cyanation.^100–102^ The BIOX®^103^ and Bacox™ processes^104^ are examples of this and are estimated to generate around 5% of global gold production.^105^ Two general types of organisms are used (i) chemolithotrops that use Fe sulfides as an energy source, producing sulfuric acid that leaches metals and (ii) cyanobacteria and fungi that produce cyanide that leaches gold as [Au(CN)_2_]^−^ for which recovery follows conventional methods.

Recent reports on the treatment of waste PCBs focus on the combined bioleaching of copper and gold. A two-step process with Cu and Au recovery efficiencies of 98% and 44%, respectively was reported in which copper leaching is accomplished with the chemolithotrops *Acidithiobacillus ferrovorans* and *Acidithiobacillus thiooxidans* followed by gold leaching using the cyanide-producing *Pseudomonas putida* under very mild operating conditions (pH 7.3–8.6 at 30 °C in 2 days).^106^ Successful gold leaching (of around 15%) from electronic waste using the cyanogenic bacterium *Chromobacterium violaceum* has also been reported,^107^ and similar findings were seen in the successful copper and gold recovery (both around 10%) from waste PCBs.^108^ As with conventional chemical leaching processes, gold recovery rates were improved if the copper was separated first; this last finding was echoed in the use of the cyanogenic bacterium *Bacillus megaterium* for gold leaching.^109^ The use of microorganisms to recover metals opens up avenues of investigation using synthetic biology. Genetically engineered strains of *Chromobacterium violaceum* with enhanced cyanide production have been created and have been shown to boost the level of gold recovery from 11% to 30%.^110^

A recent biomass adsorption process was developed for the recovery of gold and silver, along with base metals, from waste PCBs using a thiourea/sulfuric acid leachant, followed by selective adsorption on a low-cost and environmentally benign biomass gel prepared from leaf tannin.^111^ This gel was found to be more efficient at recovering gold and silver from the leached PCBs than the traditional cementation processes, and the adsorbed metals, which were reduced to their metallic form, were easily recovered by incinerating the metal-loaded gel. A high adsorption capacity bioadsorbant powder has been prepared from *Lagerstroemia speciosa* leaf tannins and polyethyleneimine, which successfully recovered gold from electronic waste and demonstrated four recyclable cycles using acidic thiourea as the eluting agent for gold recovery.^112^ Finally, the biosorption of gold from a thiourea leached liquor obtained from discarded PCBs using chitin, a fibrous polysaccharide which is chemically similar to cellulose, has been studied.^113^ In this case, *N*-acetyl and hydroxyl groups act as metal binding sites, and gold recovery rates of around 80% were observed at room temperature over a time scale of just four hours.

## Conclusions and outlook

In this review, the challenges and rewards in recovering gold from waste PCBs, which can help secure the high global demand for this valuable metal have been highlighted. Growing societal and environmental awareness of the current (often illegal) practice in collecting and reprocessing waste electronics, combined with economic drivers, will lead to greater regulation in this industry and here the substantial body of academic literature will play an instrumental part in providing routes suitable for industrial scale-up that are based on sustainable chemistry principles. Current industrial processes rely heavily on pyrometallurgy, where the high throughput, minimal pre-treatment steps, combined with ability to handle heterogeneous material, render this economically attractive. While highly energy-intensive, its reliance on fossil fuels can be partially offset by using the plastic content of PCBs as fuel. Even so, substantial challenges remain in minimising the pollution generated through incinerating plastics. Hydro- and biohydro-metallurgy offer lower capital investment routes which, along with flexibility of scale, are attractive options for both developed and developing countries alike, provided they can compete with the economy of scale offered by pyrometallurgy, deal with the challenge of the highly complex feed stream, and limit the discharge of organic chemicals into the environment. While much of the current unregulated practices draw heavily on cyanide-based hydrometallurgy processes used in primary mining operations, reports on novel leaching and extraction agents using less toxic reagents, which also address the different chemical environments presented in leaching metallic gold from PCBs, are burgeoning. Similarly, there is a wealth of literature that highlights the promise offered by biohydrometallurgy and biomass adsorption. With the potential to process low-grade material cheaply and under mild conditions, these routes are likely to make a positive impact, although life-cycle analyses would be required to fully appreciate their benefits or otherwise. However, it is clear that recovering valuable metals like gold from discarded household items such as mobile phones is a compelling and growing field, with many promising avenues arising for sustainable chemical processes.

## Conflicts of interest

There are no conflicts to declare.

## Supplementary Material
